# The Dopamine D5 Receptor Is Involved in Working Memory

**DOI:** 10.3389/fphar.2017.00666

**Published:** 2017-10-06

**Authors:** Gregory V. Carr, Federica Maltese, David R. Sibley, Daniel R. Weinberger, Francesco Papaleo

**Affiliations:** ^1^Lieber Institute for Brain Development, Baltimore, MD, United States; ^2^Department of Pharmacology and Molecular Sciences, Johns Hopkins School of Medicine, Baltimore, MD, United States; ^3^Clinical Brain Disorders Branch, Genes, Cognition and Psychosis Program, National Institute of Mental Health, National Institutes of Health, Bethesda, MD, United States; ^4^Department of Neuroscience and Brain Technologies, Istituto Italiano di Tecnologia, Genova, Italy; ^5^Molecular Neuropharmacology Section, National Institute of Neurological Disorders and Stroke, National Institutes of Health, Bethesda, MD, United States; ^6^Departments of Psychiatry and Behavioral Sciences, Neurology, and Neuroscience, The McKusick-Nathans Institute of Genetic Medicine, Johns Hopkins School of Medicine, Baltimore, MD, United States

**Keywords:** dopamine, D5 dopamine receptor, working memory, prefrontal cortex, Akt, cognition, recency memory, mice

## Abstract

Pharmacological studies indicate that dopamine D_1_-like receptors (D_1_ and D_5_) are critically involved in cognitive function. However, the lack of pharmacological ligands selective for either the D_1_ or D_5_ receptors has made it difficult to determine the unique contributions of the D_1_-like family members. To circumvent these pharmacological limitations, we used D_5_ receptor homozygous (-/-) and heterozygous (+/-) knockout mice, to identify the specific role of this receptor in higher order cognitive functions. We identified a novel role for D_5_ receptors in the regulation of spatial working memory and temporal order memory function. The D_5_ mutant mice acquired a discrete paired-trial variable-delay T-maze task at normal rates. However, both $D_{5}^{+/-}$ and $D_{5}^{-/-}$ mice exhibited impaired performance compared to $D_{5}^{+/+}$ littermates when a higher burden on working memory faculties was imposed. In a temporal order object recognition task, $D_{5}^{+/-}$ exhibited significant memory deficits. No D_5_-dependent differences in locomotor functions and interest in exploring objects were evident. Molecular biomarkers of dopaminergic functions within the prefrontal cortex (PFC) revealed a selective gene-dose effect on Akt phosphorylation at Ser473 with increased levels in $D_{5}^{-/-}$ knockout mice. A trend toward reduced levels in CaMKKbeta brain-specific band (64 kDa) in $D_{5}^{-/-}$ compared to $D_{5}^{+/+}$ was also evident. These findings highlight a previously unidentified role for D_5_ receptors in working memory function and associated molecular signatures within the PFC.

## Introduction

Dopaminergic signaling in the brain serves a critical role in cognitive functions (Nieoullon, 2002; Papaleo et al., 2008, 2012; Detrait et al., 2016). This is especially evident in higher order executive functions modulated by the prefrontal cortex (PFC) such as attentional control, working memory, cognitive flexibility, and decision-making (Robbins and Arnsten, 2009; Floresco, 2013; Papaleo et al., 2014). In particular, consistent evidence indicates that the mesocortical dopaminergic system modulates these different cognitive processes by distinct receptor mechanisms. Specifically, activity of the D_1_-like (D_1_ and D_5_) receptor family has a strong impact on the regulation of working memory, attention, and recency memory across multiple species (Sawaguchi and Goldman-Rakic, 1994; Müller et al., 1998; Aultman and Moghaddam, 2001; Lidow et al., 2003; Managò et al., 2016). In contrast, both D_1_-like and D_2_-like (D_2_, D_3_, D_4_) receptor families seem to be implicated in mediating the ability to shift between attentional sets (i.e., cognitive flexibility) (Floresco et al., 2006). Unfortunately, currently available D_1_-like agonists and antagonists do not have significant selectivity for either the D_1_ or the D_5_ receptors (Nichols, 2010). Moreover, in the cortex there is significant overlap between D_1_ and D_5_ receptor localization, and the D_1_ receptor is much more prevalent compared to the D_5_ receptor (Smiley et al., 1994; Khan et al., 2000), further impeding the investigation of the selective role of D_5_ receptors in cortex-dependent cognitive functions.

The generation of D_1_ and D_5_ genetically modified mice has helped elucidate critical functions of the two receptors in multiple physiological processes (Smith et al., 1998; Miyamoto et al., 2001; Montague et al., 2001; Hollon et al., 2002; Karlsson et al., 2008). In particular, D_1_ receptor null mutants have deficits in higher order cognitive functions such as working memory (Drago et al., 1994; Xu et al., 1994; Holmes et al., 2001; Xing et al., 2012). In contrast, there have been fewer studies on the behavioral effects of selective disruption of the D_5_ receptor. An early study of D_5_ knockout mice indicated that the behavioral consequences of the mutation were minimal. These mice showed no alterations in general health, sensory abilities, neurological reflexes, locomotor activity and coordination, prepulse inhibition, anxiety-like states measured with the elevated plus maze and light-dark box (Holmes et al., 2001). In cognitive function, D_5_ knockout mice were first reported to have no alterations in performing the hippocampal-dependent Morris water maze or fear conditioning (Holmes et al., 2001). However, a more recent study using mice with the same mutation found significant deficits in object recognition memory, object location memory, Morris water maze performance, and reduced locomotor activity (Moraga-Amaro et al., 2016). The discrepancies between these two studies may be due to differences in the experimental procedures or the different genetic backgrounds used (Holmes et al., 2001: F2 129/SvJ1 X C57BL/6J; Moraga-Amaro et al., 2016: C57BL/6J). Additionally, there is still no information on how D_5_ receptor disruption affects PFC-dependent cognitive function such as spatial working memory and recency memory. The goal of the present study was to investigate the potential involvement of the D_5_ receptor in working memory function using a well-validated discrete paired-trial variable-delay non-match to place T-maze task (Papaleo et al., 2008, 2012) and a temporal order object recognition task (Managò et al., 2016; Papaleo et al., 2016). Both tasks have been shown to rely on medial PFC functioning (Kellendonk et al., 2006; Barker et al., 2007) and are sensitive to dopaminergic modulation (Hotte et al., 2005; Papaleo et al., 2012; Managò et al., 2016).

We show that partial reduction ($D_{5}^{+/-}$) as well as the complete absence ($D_{5}^{-/-}$) of D_5_ receptors produces working memory and recency memory deficits suggesting a previously undetected direct role for D_5_ receptors in PFC-dependent higher order cognitive functions. Finally, we unraveled subtle, but selective alterations in molecular biomarkers within the mPFC of D_5_ knockout mice. These initial data identify a previously unknown role for the dopamine D_5_ receptor in cognition and related PFC functioning.

## Materials and Methods

### Mice

The $D_{5}^{-/-}$ and their $D_{5}^{+/+}$ and $D_{5}^{+/-}$ littermates were produced as previously described (Holmes et al., 2001). The mice from this mutant line were backcrossed with C57BL/6 mice for 10 generations before testing. We utilized a heterozygous breeding scheme in order to produce mixed litters with all three genotypes. Mouse genotypes were confirmed by PCR. Mice were weaned at P28 and group housed except in the T-maze experiments where mice were single housed starting 1 week before testing. Mice used for testing were male and between P63 and P126 days of age. All procedures were approved by the National Institute of Mental Health Animal Care and Use Committee and followed the National Institutes of Health *Using Animals in Intramural Research* Handbook.

### Discrete Paired-Trial Variable-Delay T-Maze Task

The procedure for this T-maze task was similar to one previously used in our laboratory (Papaleo et al., 2008). Mice were habituated to single housing for 1 week and were then food restricted to a level of 85% of their free-feeding weight. The mice were given 8 days for their weight to stabilize and received access to 10 reward pellets (5TUL 14 mg pellets; TestDiet, Richmond, IN, United States) during the last 3 days of this period. Following habituation to single housing and stabilization of body weight, mice were habituated to the T-maze apparatus over the course of two sessions. The T-maze apparatus was made of clear acrylic [dimensions of arms (length × width × height): 40 × 10.2 × 17.5 cm]. A recessed food cup was located at the end of each arm. During habituation sessions mice were allowed to retrieve reward pellets from the food cups. At the beginning of the session, each cup was baited with two reward pellets. The cups were re-baited continuously. Mice were allowed to retrieve 16 reward pellets during Session 1 and 20 reward pellets during Session 2. Each session automatically ended after 10 min if the mouse did not retrieve the maximum number of reward pellets. On the day following habituation, mice were given one session of 10 forced-alternation runs. For this session, one goal arm was blocked and the mouse had 2 min to consume the reward pellet located in the open arm. After an inter-trial interval of at least 15 min, the mouse was returned to the maze for another forced run with the open/closed arms switched. Training for the discrete paired-trial delayed alternation task began on the following day. Training consisted of 10 paired trials each day. A paired trial consisted of a forced run where one arm was blocked and the other arm was baited with a single reward pellet. The mouse was given 4 min to consume the pellet. Following consumption, the mouse was returned to the home cage for a 4-s intratrial delay. After the intratrial delay, the mouse was returned to the maze with access to both arms. The arm blocked on the forced run was now baited with two reward pellets. Again, the mouse was given 4 min to consume the reward pellets. After an inter-trial interval of at least 15 min, mice were returned for another trial. If the mouse entered the unbaited arm, this was recorded as an error and the mouse was removed from the maze. The normal inter-trial interval followed incorrect trials as well. Each testing session utilized a pseudo-randomly chosen pattern of 10 forced runs. Each day, the same pattern was used for each mouse. Mice were trained using these parameters for 20 days or until they reached 80% accuracy for 3 consecutive days. Mice that failed to reach 80% accuracy for 3 consecutive days within the 20-day training period were excluded from the study. Mice were then tested using variable intratrial intervals (4, 30, 60, and 240 s) and a 20-s inter-trial interval. Mice were given four trials of each inter-trial interval on 4 consecutive days.

### Open Field Locomotor Activity

The experimental apparatus consisted of a novel Plexiglas open field arena (42 × 42 × 30 cm) under red light illumination (5 ± 2 lux). Each mouse was allowed to freely explore the open field alone for 60 min. Horizontal locomotor activity was recorded using infrared photobeam sensors and the VersaMax Open Field Activity Monitoring system (AccuScan Instruments, Inc., Columbus, OH, United States).

### Temporal Order Object Recognition Task

Temporal order object recognition testing was conducted as previously described (Managò et al., 2016). The apparatus and lighting conditions were identical to those used for the open field locomotor activity test. On day 1, mice were allowed to freely explore the open field for 60 min. Day 2 consisted of three 5-min sessions. During the first session, mice were allowed to explore two identical objects within the open field arena. The objects were either rectangular boxes (3 × 3 × 6 cm) or Erlenmeyer flasks (4 × 6 cm). The objects could either be white or black. During the second session, 1 h after the first session, mice were allowed to explore two objects of a different shape and color with respect to the objects from the first session. During the third session, 3 h after the second session, mice were allowed to explore one copy of each of the objects presented during the first and second sessions. The sessions were videotaped and scored offline by a reviewer blind to genotype. Mice were considered to be exploring an object when they faced the object and were ≤2 cm from the object. Discrimination between the objects in the third session was calculated using a discrimination index that accounts for individual differences in total exploration time. The index was calculated as the difference between the time spent exploring the object from the first session and the object from the second session divided by the total exploration time. Any mice that did not explore objects for more than 4 s during all of the sessions were excluded from the final analysis. One $D_{5}^{+/+}$, one $D_{5}^{+/-}$, and four $D_{5}^{-/-}$ mice were excluded because of low total exploration.

### Immunoblotting

Frontal cortex tissue was obtained from naive $D_{5}^{+/+}$, $D_{5}^{+/-}$, and $D_{5}^{-/-}$ mice. Briefly, mice were killed by decapitation and the brain was removed and placed on a glass tray on ice. The olfactory bulbs were removed and the brain was cut along the midline. The frontal cortex (anterior to the corpus callosum) was removed and flash frozen on dry ice. The tissue was then stored at -80°C until processing. The tissue was then homogenized and sonicated in T-Per (Thermo Scientific, Rockford, IL, United States) lysis buffer. The protein concentration of the samples was determined by Bradford assay and all samples were diluted to a final concentration of 2 μg/μl. The samples were then combined with NuPage^®^ LDS Sample Buffer (ratio of 3:1 sample:LDS buffer). The protein was then denatured by heating at 95°C for 5 min. Samples were then run on pre-cast 4–12% Bis-Tris gels. After transfer to PVDF membranes, blots were blocked with 5% milk in TTBS for 1 h at room temperature. We probed the blots with the primary antibodies at 4°C overnight (**Table 1**). Following three 10-min washes in TTBS, blots were probed with the goat anti-rabbit, goat anti-mouse (1:10,000 dilution; Chemicon, Temecula, CA, United States), or donkey anti-goat (1:1000; Santa Cruz Biotechnology, Dallas, TX, United States) secondary antibodies at room temperature for 1 h. Blots were developed in ECL-Plus (GE Healthcare, Piscataway, NJ, United States) and exposed to Kodak Bio-Max film. Films were digitized using a scanner, and the resulting images were analyzed using NIH Image gel plotting macros.

**Table 1:** Primary antibodies.

Antigen	Type (clone)	Dilution	Product number	Manufacturer
pAkt (Thr308)	RbM (C31E5E)	1:1000	2965	Cell Signaling Technology
pAkt (Ser473)	RbP	1:1000	9271	Cell Signaling Technology
tAkt	MM (40D4)	1:2000	2920	Cell Signaling Technology
Camkkbeta	GP	1:1000	sc-9629	Santa Cruz Biotechnology
Camk2	MM (G-1)	1:5000	sc-5306	Santa Cruz Biotechnology
Camk4	GP	1:200	sc-1541	Santa Cruz Biotechnology
Drd2	MM (B-10)	1:1000	sc-5303	Santa Cruz Biotechnology
Comt	MM (4/COMT)	1:10,000	611970	BD Biosciences
pTH Ser40	RbP	1:800	AB5935	Millipore
TH	RbP	1:2000	AB 152	Millipore
GAPDH	MM (mAbcam 9484)	1:10,000	ab9484	Abcam
Actin	RbP	1:5000	A2066	Sigma–Aldrich

*RbM, rabbit monoclonal; RbP, rabbit polyclonal; MM, mouse monoclonal; GP, goat polyclonal.*

### Statistical Analysis

The habituation and training phases of the T-maze task were analyzed using one-way ANOVAs with *post hoc* Bonferroni’s tests. The variable delay portion was analyzed by a two-way ANOVA with genotype as the between-subjects factor and retention interval as the within-subjects factor and *post hoc* analyses utilized Bonferroni’s tests at each of the retention intervals. Data from the temporal order object recognition task were analyzed using a one-way ANOVA with *post hoc* Bonferroni’s tests. Protein quantification data for each mouse was first normalized to GAPDH levels (actin for TH protein levels) and then normalized to the mean $D_{5}^{+/+}$ value for each protein. Group means were then compared using one-way ANOVAs with *post hoc* Bonferroni’s tests. All data are shown as the mean ± SEM.

## Results

### D_5_ Genetic Disruption Did Not Alter Locomotor Functioning or Approach Responses to Food Reward

**FIGURE 1:**
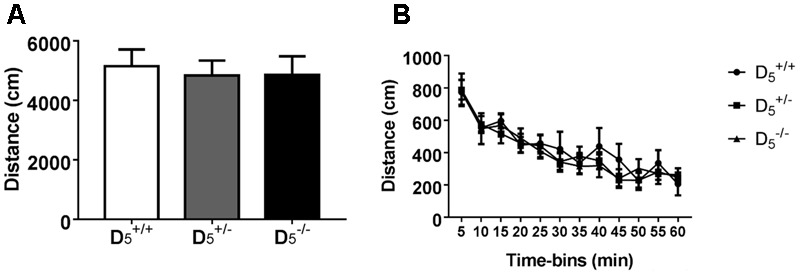
Locomotor activity is normal in $D_{5}^{+/-}$ and $D_{5}^{-/-}$ mice. No difference between genotypes in either **(A)** total locomotor activity or **(B)** individual 5-min time bins. *n* = 6 in the $D_{5}^{+/+}$ group, 16 in the $D_{5}^{+/-}$ group, and 11 in the $D_{5}^{-/-}$ group.

To identify any potential confounding effects of D_5_ receptor disruption, we measured open field locomotor activity and approach responses to food reward in the T-maze apparatus. There were no D_5_ genotype differences in total locomotor activity (*F*_(2,30)_ = 0.06, *p* = 0.9420; **Figure 1A**) or any genotype × time interactions during any of the 5-min time bins within the 60-min test (*F*_(22,330)_ = 0.63, *p* = 0.9000; **Figure 1B**). As expected there was a significant decrease in activity over time for all groups (*F*_(11,330)_ = 35.68, *p* < 0.0001; **Figure 1B**).

**FIGURE 2:**
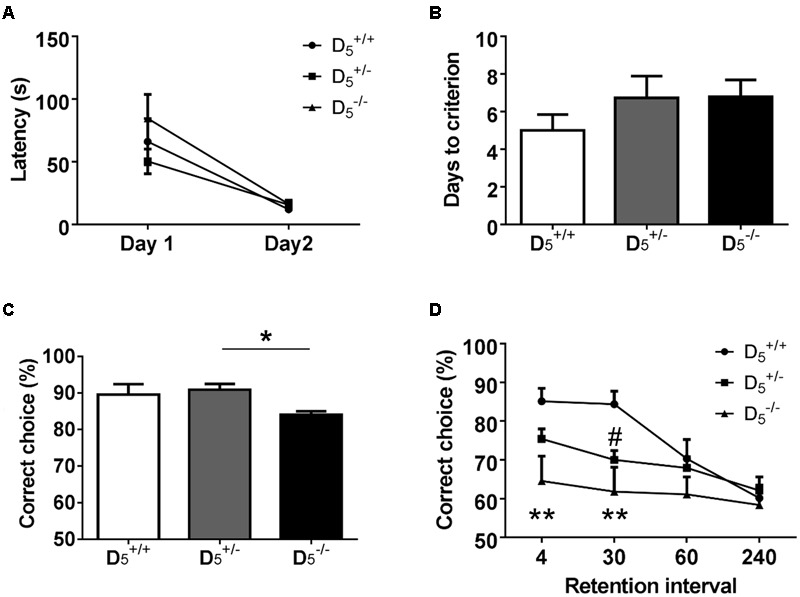
$D_{5}^{+/-}$ and $D_{5}^{-/-}$ mice display spatial working memory deficits. **(A)** During habituation, there are no genotype differences in latency to consume the first pellet. **(B)** All mice learn the non-match to sample rule in the same number of days. **(C)** $D_{5}^{-/-}$ mice had significantly lower accuracy compared to $D_{5}^{+/-}$ mice, but were no different from $D_{5}^{+/+}$ mice on the last 3 days of training. **(D)** During testing when the inter-trial interval was decreased to 20 s, $D_{5}^{-/-}$ mice showed decreased performance at the 4 and 30-s retention intervals compared to $D_{5}^{+/+}$ mice. Additionally, $D_{5}^{+/-}$ mice demonstrated impaired performance at the 30-s retention interval. *n* = 8 in the $D_{5}^{+/+}$ group, 15 in the $D_{5}^{+/-}$ group, and 9 in the $D_{5}^{-/-}$ group. ^∗^*p* < 0.05, ^∗∗^*p* < 0.01 ($D_{5}^{-/-}$ compared to $D_{5}^{+/+}$) and ^#^*p* < 0.05 ($D_{5}^{+/-}$ compared to $D_{5}^{+/+}$).

The first phase (two sessions) of the T-maze task is designed to habituate the mice to the testing apparatus and retrieval response required for completion of the task. All groups showed a significant decrease in latency to consume the first pellet from Day 1 to Day 2 (*F*_(1,30)_ = 41.71, *p* < 0.0001; **Figure 2A**). Additionally, there were no differences in the raw latency values between the genotypes on either Day 1 or Day 2 (*F*_(2,30)_ = 1.20, *p* = 0.31). These data support the notion of a negligible impact of D_5_ on measures of locomotion and motivation.

### D_5_ Knockout Mice Learn the Non-match to Sample Rule at the Same Rate As $D_{5}^{+/+}$ Mice

The next stage of the T-maze task consisted of training required for the mice to learn the non-match to sample rule. There were no significant differences in the number of sessions required to reach the criterion of three consecutive sessions above 80% accuracy (*F*_(2,29)_ = 0.70, *p* = 0.50; **Figure 2B**). However, there was a significant difference in accuracy during those last three training sessions (*F*_(2,29)_ = 3.63, *p* = 0.04; **Figure 2C**). The genotype effect on accuracy was driven by a slight, but significant, difference between $D_{5}^{+/-}$ and $D_{5}^{-/-}$ (*p* = 0.04). The difference between $D_{5}^{+/+}$ and $D_{5}^{-/-}$ was not significant (*p* = 0.22). These data indicate a marginal role of the D_5_ receptor in the ability to acquire working memory rules.

### D_5_ Knockout Mice Have Working Memory Deficits Compared to $D_{5}^{+/+}$ When Tested in the Variable Retention Version of the Task

Following successful completion of the training phase, mice were tested in the variable retention delay portion of the task. In addition to the variable retention delays, the inter-trial delay was set at 20 s (Aultman and Moghaddam, 2001). Accuracy decreased as the retention interval increased across all genotypes (*F*_(2,29)_ = 11.00, *p* < 0.0001). There was also a significant main effect of D_5_ genotype on performance (*F*_(2,29)_ = 4.91, *p* = 0.01). $D_{5}^{-/-}$ mice showed significantly impaired performance compared to $D_{5}^{+/+}$ on trials with either 4- or 30-s retention intervals (*p* = 0.004 and 0.001, respectively; **Figure 2D**). $D_{5}^{+/-}$ mice had impaired performance at the 30-s interval compared to $D_{5}^{+/+}$ mice (*p* = 0.033; **Figure 2D**). There were no significant differences between the genotypes on either the 60- or 240-s retention intervals due to a decrease in the choice accuracy of the $D_{5}^{+/+}$ mice. Interestingly, the $D_{5}^{+/-}$ mice had an intermediate phenotype suggesting that there is a gene dose effect of D_5_ dopamine receptor expression. These findings highlight a clear and previously undetected role of D_5_ receptors in working memory abilities.

### $D_{5}^{+/-}$ Mice Have Recency Memory Deficits

**FIGURE 3:**
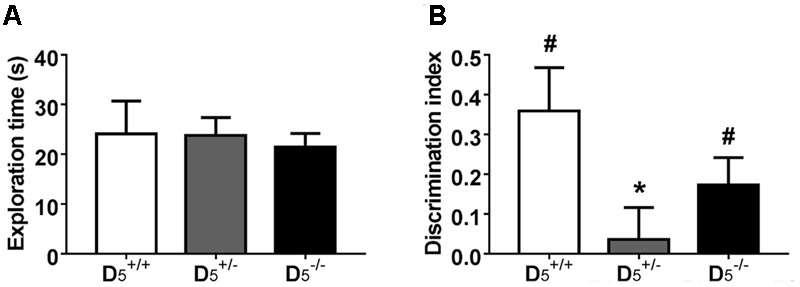
$D_{5}^{+/-}$ mice have impaired temporal order memory. **(A)** The differences in the recognition index were not confounded by any differences in total exploration time during the test phase. **(B)** Unlike $D_{5}^{+/+}$ and $D_{5}^{-/-}$, $D_{5}^{+/-}$ mice have no preference for the object presented during sample phase 1, indicating deficits in temporal order memory. *n* = 8 in the $D_{5}^{+/+}$ group, 15 in the $D_{5}^{+/-}$ group, and 11 in the $D_{5}^{-/-}$ group. ^∗^*p* < 0.05 compared to $D_{5}^{+/+}$ and ^#^*p* < 0.05 compared to hypothetical zero.

Performance in a temporal order object recognition task, like the non-match to sample T-maze task, has been shown to depend on intact PFC function (Barker et al., 2007). There were no differences in total exploration between the three genotypes (*F*_(2,31)_ = 0.12, *p* = 0.89; **Figure 3A**), but there was a significant difference in the discrimination index (*F*_(2,31)_ = 3.33, *p* = 0.049; **Figure 3B**). *Post hoc* analysis indicated that the $D_{5}^{+/-}$ group had a significantly lower discrimination index compared to the $D_{5}^{+/+}$ group (*p* = 0.046), but only a tendency was evident for $D_{5}^{-/-}$ mice. Thus, there might be an U-shaped gene-dose effect on temporal order object recognition as the $D_{5}^{-/-}$ group’s discrimination index was not different from the $D_{5}^{+/+}$ group’s index (*p =* 0.52). One sample *t*-tests indicated that both the $D_{5}^{+/+}$ (*t*_(7)_ = 3.28, *p* = 0.01) and $D_{5}^{-/-}$ (*t*_(10)_ = 2.50, *p* = 0.03) groups showed significant recency memory while the $D_{5}^{+/-}$ (*t*_(14)_ = 0.44, *p* = 0.67) group did not.

### D_5_ Knockout Mice Show Selective Gene-Dose Effect on Akt Ser473 Phosphorylation in the PFC

The working memory and temporal order recognition deficits exhibited by D_5_ mutant mice are similar to those our group has observed in other mouse genetic models of dopamine-related cognitive dysfunction (Papaleo et al., 2008, 2012). Moreover, performance in the discrete paired-trial variable-delay T-maze and temporal order object recognition tasks has been shown to be modulated by alterations in PFC function (Kellendonk et al., 2006; Barker et al., 2007). Thus, we next investigated whether D_5_ knockout mice might have working memory- and dopamine-related molecular alterations within the mPFC (Papaleo et al., 2008, 2014; Tan et al., 2012; Easton et al., 2013; Managò et al., 2016).

**FIGURE 4:**
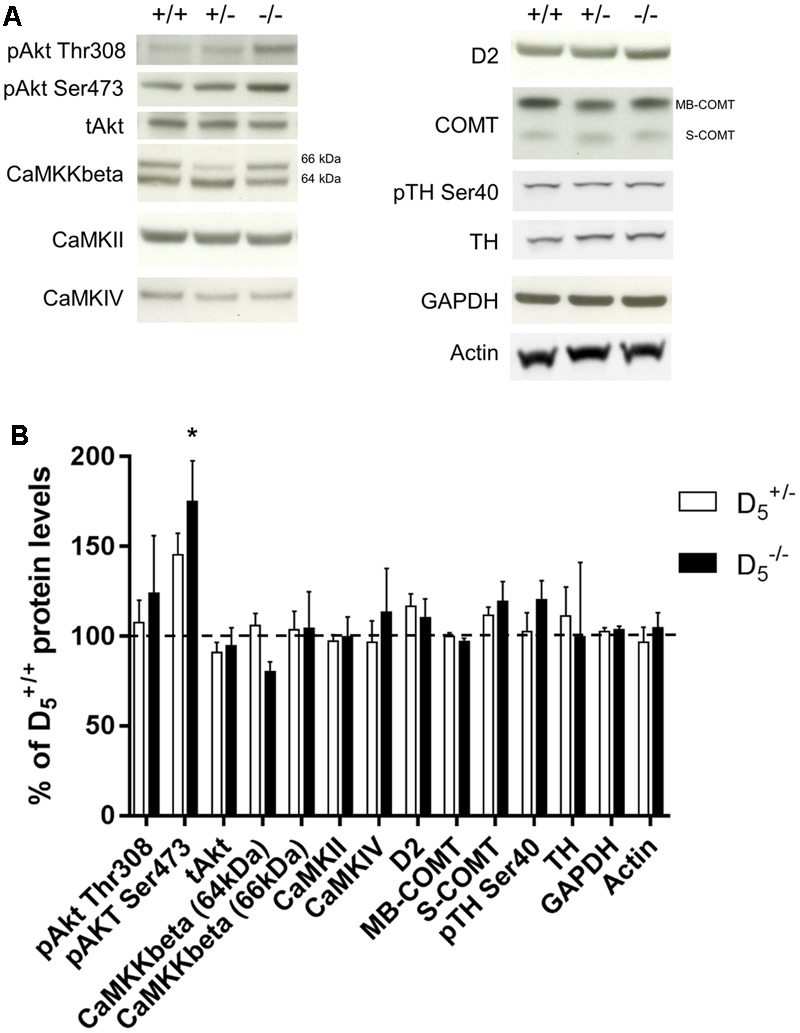
Protein levels of potential molecular substrates in prefrontal cortex. **(A)** Representative blots for each genotype. *n* = 6 in the $D_{5}^{+/+}$ group, 13 in the $D_{5}^{+/-}$ group, and 6 in the $D_{5}^{-/-}$ group for all Western blots except for CaMKKbeta ($D_{5}^{+/+}$ group = 12, $D_{5}^{+/-}$ group = 19, and $D_{5}^{-/-}$ group = 11), TH, pTH Ser40, and actin ($D_{5}^{+/+}$ group = 6, $D_{5}^{+/-}$ group = 6, and $D_{5}^{-/-}$ group = 4). **(B)** Protein levels in $D_{5}^{+/-}$ and $D_{5}^{-/-}$ mice compared to $D_{5}^{+/+}$ mice. Data are normalized to the group mean for the $D_{5}^{+/+}$ group within each protein. The dashed line represents the $D_{5}^{+/+}$ mean (100%). ^∗^*p* < 0.05 compared to $D_{5}^{+/+}$.

We measured the relative amounts of multiple proteins in the frontal cortex across all three genotypes. These data are presented in **Figure 4**. Akt is a key intracellular regulatory protein involved in dopaminergic signaling and implicated in psychiatric disorders (Emamian et al., 2004; Beaulieu et al., 2007). There was no significant difference in pAkt Thr308 (*F*_(2,22)_ = 0.46, *p* = 0.63; **Figures 4A,B**) or total Akt (*F*_(2,22)_ = 0.46, *p* = 0.63). However, there was a significant genotype effect on pAkt Ser473 protein levels (*F*_(2,22)_ = 4.90, *p* = 0.02) with $D_{5}^{-/-}$ mice exhibiting increased phosphorylation compared to $D_{5}^{+/+}$ mice (*p* = 0.02). Despite seeing large changes in CAMKKβ protein levels in other genetic mouse models with working memory deficits (Papaleo et al., 2008, 2012), there were no changes in the CAMKKβ 66 kDa (*F*_(2,22)_ = 0.03, *p* = 0.97) and a small, but not statistically significant, decrease in $D_{5}^{-/-}$ mice compared to $D_{5}^{+/+}$ on the 64 kDa (*F*_(2,22)_ = 2.315, *p* = 0.12) isoform. Protein levels of another Ca^2+^-dependent kinase CAMKII were not significantly different between the genotypes (*F*_(2,22)_ = 0.07, *p* = 0.94). Also, protein levels of the CAMKKβ substrate CAMKIV were unaffected by the D_5_ genotype (*F*_(2,22)_ = 0.31, *p* = 0.73). Finally, we measured levels of dopamine-associated proteins and found no significant differences in the D_2_ receptor (*F*_(2,22)_ = 1.19, *p* = 0.32), membrane-bound catechol-*O*-methyltransferase (COMT) (*F*_(2,22)_ = 0.65, *p* = 0.53), soluble COMT (*F*_(2,22)_ = 1.79, *p* = 0.19), phosphorylated (Ser40) tyrosine hydroxylase (TH) (*F*_(2,13)_ = 0.01, *p* < 0.99), or TH total protein levels (*F*_(2,13)_ = 1.04, *p* < 0.38; **Figures 4A,B**). Overall, these findings indicate a selective impact on PFC Akt activation by D_5_ receptor while sparing other dopamine-related biomarkers.

## Discussion

In this study, we show that disruption of the dopamine D_5_ receptor results in impaired spatial working memory and temporal order memory function. These findings unravel a previously unidentified selective involvement of the D_5_ receptor as a critical modulator of higher order cognitive functions associated with the PFC.

The lack of pharmacological agents with selectivity for either the D_1_ or D_5_ receptor has made it difficult to identify the specific contributions of either receptor to central nervous system function and behavior (Nichols, 2010). Previous research with total $D_{5}^{-/-}$ mice and conditional constructs indicated that the D_5_ receptor, in contrast to the D_1_ receptor, plays a modest role in dopamine-mediated behaviors (Holmes et al., 2001; Karlsson et al., 2008; Sariñana et al., 2014). However, recent studies utilizing $D_{5}^{-/-}$ mice suggest a role in fear memory consolidation through modulation of phospholipase C signaling (Ouyang et al., 2012) and a role in regulating BDNF and Akt function in the PFC (Perreault et al., 2013). A recent study using the same line of D_5_ mutant mice as our current study also identified deficits in spatial and recognition memory in the knockout mice (Moraga-Amaro et al., 2016). Those mice also exhibited reduced locomotor activity, reduced object exploration, and increased anxiety-related states (i.e., increased latency to explore objects), not seen by either Holmes et al. (2001) or us, that may have influenced their cognitive performance. The differences in locomotor activity and object exploration between our study and the report of Morago-Amaro and colleagues may be due to differences in experimental procedures or genetic background. Nonetheless, loss of the D_5_ receptor appears to significantly alter behavior including cognitive function. The spatial working memory deficit we describe is similar to other genetic mouse models characterized by altered dopaminergic function in the PFC (Papaleo et al., 2008, 2012). These findings indicate that dopaminergic signaling through the D_5_ receptor may serve a previously underappreciated role in behavior. Our current work is the first report indicating the involvement of the D_5_ receptor in spatial working memory function measured with a delayed non-match to place T-maze task.

Previous studies using D_1/5_ agonists and antagonists have implicated D_1_-like receptors in the regulation of working memory (Aultman and Moghaddam, 2001; Mizoguchi et al., 2009). Additionally, the D_5_ receptor is widely expressed in the cerebral cortex and hippocampus, regions critically involved in spatial working memory function (Knowlton et al., 1985; Luciana and Collins, 1997; Ciliax et al., 2000; Khan et al., 2000), suggesting there may be a specific role for the D_5_ receptor. Interestingly, there appears to be some redundancy in D_1_-like receptor modulation of working memory. Indeed, D_1_ receptor knockout mice, like D_5_ receptor knockout mice, show deficits in working memory function and abnormal regulation of BDNF in the PFC (Xing et al., 2012). Like spatial working memory, temporal order recognition memory requires intact signaling between the PFC and hippocampus (Barker et al., 2007; Barker and Warburton, 2011). Here we report a potential U-shaped relationship between the degree of D_5_ receptor insufficiency and performance in the temporal order recognition task in contrast to the apparent linear gene-dose relationship seen in spatial working memory. Although the underlying cause of the discrepancy between relative performance in the temporal order object recognition task and discrete paired-trial variable-delay T-maze is unknown, previous research has shown that the optimal dopaminergic tone is variable depending on the particular task with which the animal is currently engaged (Floresco, 2013).

In the current experiments, we investigated the protein levels of CaM kinases because previous studies using mouse models of dopaminergic dysfunction suggested a role for this family of kinases in modulating working memory in this particular T-maze task (Papaleo et al., 2012). In particular, our previous studies linked an alteration of overall dopamine levels within the PFC (Papaleo et al., 2008) or altered D_2_ trafficking (Papaleo et al., 2012) with CaM kinases expression. In contrast, no major D_5_-dependent effect was evident in CaM levels, with the possible exception of the brain-specific CaMKKβ isoform. We did not observe any alterations in TH or dopamine D_2_ receptor protein levels. Moreover, previous research demonstrated no change in dopamine D_1_ receptor function following D_5_ inactivation (Hollon et al., 2002). Thus, our findings combined with previous evidence that D_1_/D_5_ receptor pathways modulate PFC long-term potentiation and intrinsic excitability through the activation of CaMK pathways (Chen et al., 2007) suggest a possible selective role of D_1_ receptors in these processes.

The only significant change in our protein assays in the PFC resulting from the loss of the D_5_ receptor was an increase in pAkt (Ser473). This might be in agreement with previous pharmacological manipulation suggesting that the D_5_ receptor regulates phosphorylation of Akt in the PFC in mice (Perreault et al., 2013). Akt activity has been linked to cell proliferation, growth, survival, and metabolism, and it has been implicated in sex differences and psychiatric disorders (Chen et al., 2004; Emamian et al., 2004; Beg et al., 2017; Sannino et al., 2017). In particular, Akt activity has been proposed as an intracellular key regulatory protein directly linked to the activity of D_2_ postsynaptic receptors (Beaulieu et al., 2007). Here we add new evidence implicating D_5_ receptors in Akt-mediated signaling that will require further and more focused investigation.

The D_5_ receptor is uniquely located to play an important role in modulation of PFC function. Anatomical studies in nonhuman primates show that D_5_ receptors are positioned in extrasynaptic microdomains where they can interact with the 1,4,5-triphosphate receptor to mobilize calcium from intracellular stores (Paspalas and Goldman-Rakic, 2004). These microdomains are critical locations for the signaling mechanisms underlying dopaminergic volume transmission in the cortex. The current results point specifically to a critical role for the D_5_ receptor in PFC-dependent spatial working memory as well as recency memory. Further studies may serve to define the parameters of D_5_ dopamine receptor activity as it relates to other cognitive domains. However, given the relatively precise localization of D_5_ receptors, a therapeutic strategy selectively targeting them may improve cognitive function with potentially fewer side effects compared to drugs selectively targeting the D_1_ receptor. This would be relevant for many neurological and psychiatric disorders, such as schizophrenia, bipolar disorders, and others (Wu et al., 2012; Narayanan et al., 2013; Laruelle, 2014).

## Conclusion

$D_{5}^{+/-}$ and $D_{5}^{-/-}$ mice have spatial working memory deficits in a discrete paired-trial variable-delay T-maze task as well as recency memory deficits in a temporal order object recognition task. These data represent new evidence that the dopamine D_5_ receptor is directly involved in higher order cognitive functions.

## Author Contributions

GC, FM, DS, DW, and FP contributed to the conception and design of the reported studies. GC and FM conducted all of the experiments. GC, FM, and FP analyzed the data. GC, FM, DS, DW, and FP contributed to the drafting and revision of the manuscript. All authors approved the final version and agreed to be accountable for all aspects of the work.

## Conflict of Interest Statement

The authors declare that the research was conducted in the absence of any commercial or financial relationships that could be construed as a potential conflict of interest.
